# Parameter-Free Matrix Decomposition for Specular Reflections Removal in Endoscopic Images

**DOI:** 10.1109/JTEHM.2023.3283444

**Published:** 2023-06-06

**Authors:** Jithin Joseph, Sudhish N. George, Kiran Raja

**Affiliations:** Department of Electronics and Communication EngineeringNational Institute of Technology at Calicut 72899 Kozhikode 673601 India; Department of Computer ScienceNorwegian University of Science and Technology 8018 7034 Trondheim Norway

**Keywords:** Specular reflections, singular value thresholding, low rank and sparse decomposition

## Abstract

*Objective:* Endoscopy is a medical diagnostic procedure used to see inside the human body with the help of a camera-attached system called the endoscope. Endoscopic images and videos suffer from specular reflections (or highlight) and can have an adverse impact on the diagnostic quality of images. These scattered white regions severely affect the visual appearance of images for both endoscopists and the computer-aided diagnosis of diseases. Methods & Results: We introduce a new parameter-free matrix decomposition technique to remove the specular reflections. The proposed method decomposes the original image into a highlight-free pseudo-low-rank component and a highlight component. Along with the highlight removal, the approach also removes the boundary artifacts present around the highlight regions, unlike the previous works based on family of Robust Principal Component Analysis (RPCA). The approach is evaluated on three publicly available endoscopy datasets: Kvasir Polyp, Kvasir Normal-Pylorus and Kvasir Capsule datasets. Our evaluation is benchmarked against 4 different state-of-the-art approaches using three different well-used metrics such as Structural Similarity Index Measure (SSIM), Percentage of highlights remaining and Coefficient of Variation (CoV). Conclusions: The results show significant improvements over the compared methods on all three metrics. The approach is further validated for statistical significance where it emerges better than other state-of-the-art approaches.*Clinical and Translational Impact Statement—*The mathematical concepts of low rank and rank decomposition in matrix algebra are translated to remove specularities in the endoscopic images The result shows the impact of the proposed method in removing specular reflections from endoscopic images indicating improved diagnosis efficiency for both endoscopists and computer-aided diagnosis systems

## I. Introduction

Endoscopic procedures are used for the diagnosis of various pathologies in the internal human body with the help of a camera system. The images and videos obtained from the endoscopic procedure are used to detect any kind of abnormalities present in the examined organ through visual interpretation by the endoscopist or sometimes by a computer-aided diagnosis (CAD) system. If the images captured by the camera system contain undesired artifacts, the identification of abnormalities becomes difficult and challenging. Robust and reliable identification of abnormalities has become a fundamental medical imaging problem and is extensively studied by researchers [1], [2], [3]. A set of works focus on resolving these artifacts that originate at the time of image acquisition [4] and some during the transmission [5] and compression stages [6], [7].

**FIGURE 1.**
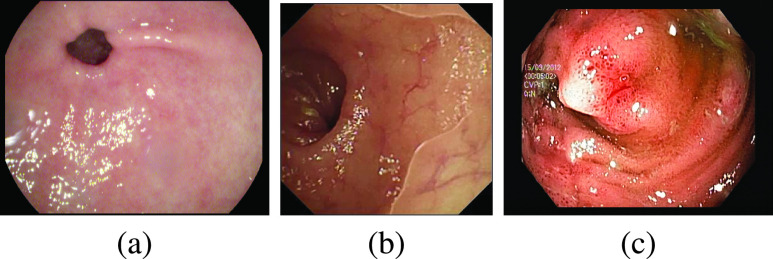
Illustration of specular reflection in three different endoscopy datasets (a) Kvasir Normal-Pylorus dataset [14], (b) Kvasir Capsule dataset [15] and (c) Kvasir Polyp dataset [14].

Six artifacts from image acquisition [4] are identified as potential challenges to detect pathologies and these include, a) existence of specular reflections, b) the presence of bubbles, c) the blurring of images, d) over-exposed pixels, e) under-exposed pixels, and f) the presence of debris and chromatic aberrations. Out of these, the identification of pathologies is severely hampered by the presence of specular reflections according to earlier studies [8], [9], [10], [11]. Usually, the watery and smooth surface of the human organs can produce specular reflections when illuminated from endoscopes. The light incident on the body surface undergoes both diffuse and specular reflections [12] due to the complex characteristics of the organ surface. The diffuse reflection component resembles the characteristics of the body surface while the specular reflection component will have the characteristics of illuminant light [13]. Fig. 1 shows an illustration of specular reflections in endoscopic images from publicly available datasets [14], [15]. One can note the specular reflections as scattered white spots impacting the overall quality of an image.

Such presence of highlights has been reported to result in failure for feature extraction, especially in surgical navigation systems that use augmented reality (AR) [8], [9], [10], [11]. This undesirable artifact may further impair the surgeon’s ability to observe and make decisions on pathology. Removing specular reflections from endoscopic images, therefore, is of primary concern to provide medical professionals with better quality images and to devise better-automated diagnosis systems.

In this paper, we present a novel approach for medical imaging applications that utilize mathematical principles of matrix decomposition and low-rank structure. Specifically, we propose the use of low-rank decomposition and singular value thresholding operations to effectively remove specular reflections from endoscopic images. Our method offers a promising solution for improving the quality and visibility of endoscopic imaging in medical diagnosis and treatment.

In the rest of the paper, previous works related to specular reflection removal are discussed in Section II. In Section III, a detailed analysis of highlight images and characteristics of highlight pixels are discussed. These analyses are used in Section IV to develop the algorithm to remove the highlights. In Section V, the results from the experiments to evaluate the efficacy of the proposed algorithm are presented along with results from state-of-the-art approaches. We then provide some concluding remarks in Section VI.

## II. Related Works and Our Contributions

Different works have been proposed to remove specular reflections from medical images. Many of them consider the reflection removal as a two-stage problem [4], [16] [17]. In the first stage, highlight pixels are discovered, and in the second stage, they are either eliminated or replaced with approximated original values. In [16], the segmentation of specular regions is based on non-linear filtering and color image thresholding. In [17], a specular lobe is identified at the tail end of the histogram for thoracoscopic images and is extracted to obtain the highlight pixel map. Kim et al. [18] proposed using geometric characteristics such as the shape of the specular region to detect highlight pixels. With the aid of a thresholding operation, highlight pixels are identified in [4] and [19] using chromatic information.

The highlight areas are scattered/dispersed throughout the entire image, as shown in Fig. 1. These specular reflection components include acute discontinuities towards the edge of the region and abrupt variations when compared to the diffusion component of reflections. Yang et al. [20], suggested a filter-based approach to eliminate unwanted specular reflection and high-frequency components by using an edge-preserving low-pass filter. However, the method is not applicable in the instances where the highlight regions create a continuous band of pixels rather than being dispersed. As a result, edge-preserving low-pass filters cannot remove the highlight pixels efficiently on all images.

In [19], [21], and [22] each image pixel is assumed to consist of two components rather than assigning individual pixels to a single component as a highlight or non-highlight component. Corresponding to each pixel, it is assumed that light gets reflected both as diffuse reflection and specular reflection. Diffuse reflection assumes the color of the tissue, and specular reflection assumes the color of the illuminant light [13]. If the specular reflections are identified using the properties of the illuminant light, then the diffuse reflection component can provide a highlight-free image. However, the surface properties of organs and the motion of the camera in the dynamic environment within the body make the approaches mentioned [19], [21], and [22], not fully practical.

**FIGURE 2.**
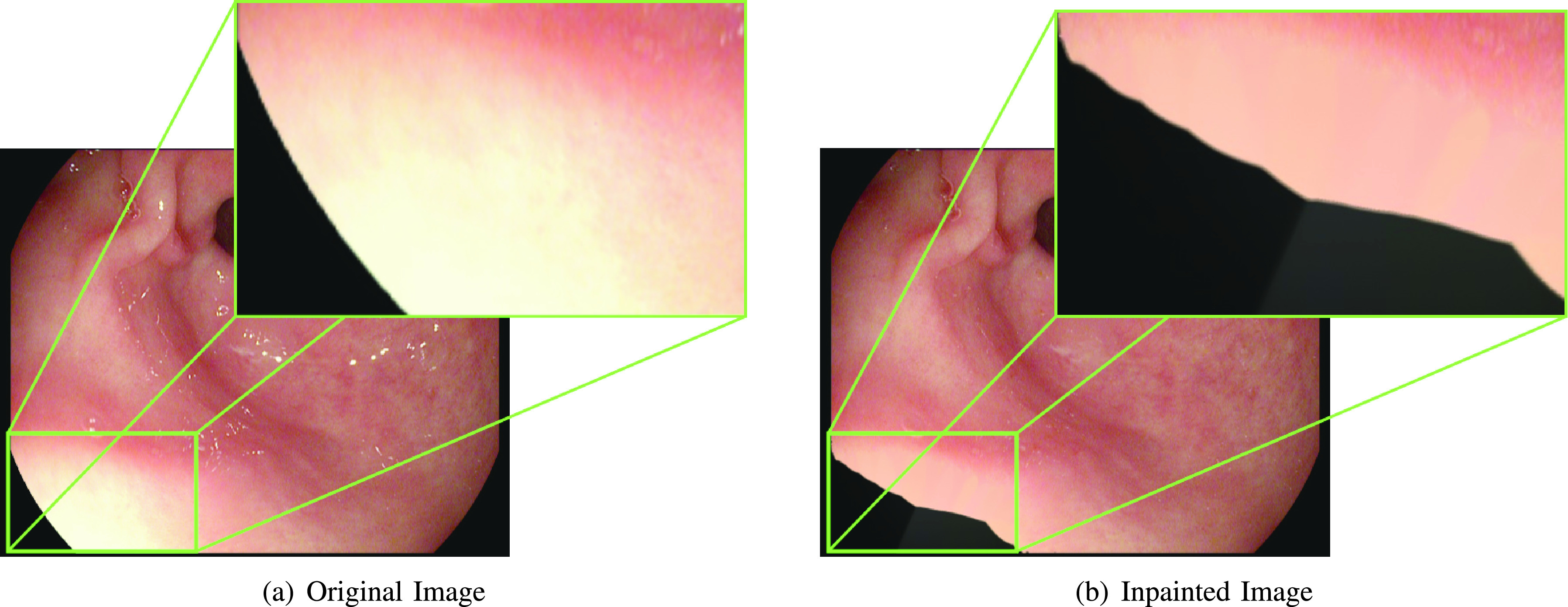
Effect of Inpainting method [16] on original image.

Arnold et al. [16] used an inpainting method to remove the highlight pixels preceded by automatic segmentation of highlight pixels. The approach ignores the global nature of the image since it uses the local information surrounding the highlight zone to inpaint the segmented sections. Even though the method is successful in reproducing segmented parts accurately, regions close to the edge of the original image are destroyed if a highlight region is in the neighborhood. Fig. 2 shows the negative effect of local inpainting [16] on an image with specular reflection near the edge. A structural similarity-based inpainting technique is also suggested by Gao et al. [24].

The deep learning, supervised and semi-supervised methods are also investigated for specular reflection removal from both natural and medical images when labeled datasets are available to train the models [25], [26] [27]. Bobrow et al. [26] used pairs of coherent and incoherent images for training. They proposed a deep learning network called DeepLSR to remove laser speckles from coherent illuminated images. The incoherent light-emitting diodes are used as the ground truth images. Funke et al. [27] proposed to use two GANs for self-training and self-regularization. The Conditional Generative Adversarial Network (cGAN) considers removing the specularity as a translation from image to image. The SpecGAN proposed in [27], trains the network from weakly labelled training data.

**FIGURE 3.**
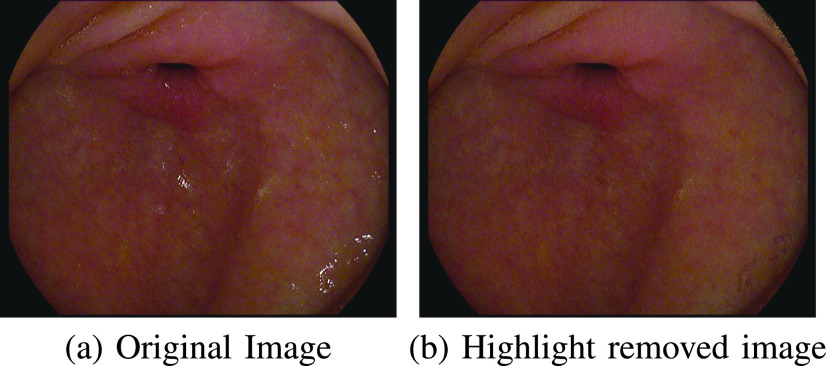
Effect of applying Adaptive RPCA [23].

**FIGURE 4.**
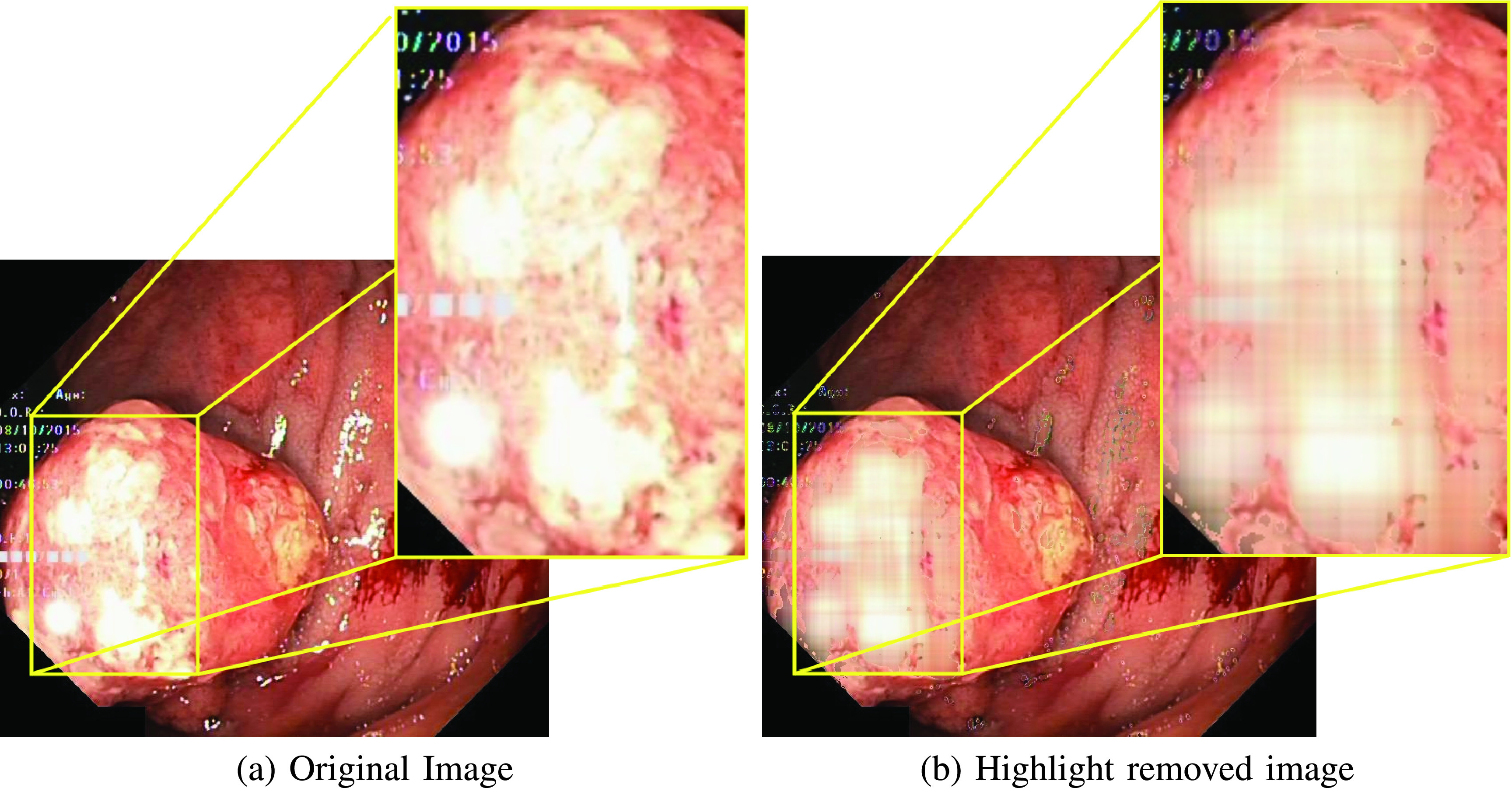
Effect of applying Adaptive RPCA [23] when the highlight is not completely sparse in nature. Images are from the Kvasir Polyp dataset.

In the absence of labelled datasets (i.e., ground-truth), specular reflection removal is carried out using the family of classical matrix decomposition methods such as Robust Principal Component Analysis (RPCA) [23], [28]. In these approaches, the highlight region is considered to be sparse in nature and by removing these sparse components, a highlight-free image is obtained, as shown in Fig. 3. However, the highlight component is not completely sparse in nature and contributes significantly to the low-rank portion of the image as shown in Fig. 4(a). As a result, the low-rank component contains some highlight. Li et al. [23] used an iterative method to set the parameters of RPCA, which itself is an iterative algorithm. However, most of the relevant information in the case of endoscopy lies in the sparse information of the image. Removing the sparse component removes this vital information from the image and further reduces the robustness of faithfully reproducing a highlight-free image. A close observation of the images from the Kvasir Polyp, Kvasir Capsule, and Kvasir Normal-Pylorus datasets reveals the presence of dark boundary regions around the highlight pixels which need to be eliminated along with highlights. The algorithm in [23] does not consider these dark boundary artifacts while reconstructing the image. Fig. 4 provides an illustration of deficiencies of specular reflection removal using RPCA based approach when the highlight is not fully sparse in nature.

### A. Our Contributions

We propose an approach based on the matrix decomposition method to address the drawbacks of the existing techniques for specular reflection removal in endoscopic images. Our major contributions are listed below:

1) We propose a new approach which exploits the characteristics of highlight pixels with an iterative decomposition procedure to generate a pseudo-low-rank component and a highlight component irrespective of the degree of sparsity. Unlike normal RPCA-based algorithms that fail to eliminate the highlight efficiently, our approach can remove the highlights even when the highlight is distributed sparsely in the image.
2) We propose to exploit human vision-based Hue-Saturation-Value (HSV) [29] color space to identify the highlight areas mimicking human vision to determine the areas of highlight. The variation of highlight distribution in the image has a direct impact on HSV space and can be used for estimating soft thresholds as given in Eq. (1) in Section IV further.
3) Our approach eliminates parameter estimation by exploiting the characteristics of highlight pixels making it a parameter-free approach unlike previous approaches. Previous unsupervised methods of highlight detection rely highly on the setting up of its parameters according to the dataset or even according to the lighting conditions of individual images [20], [23]. Unlike the earlier works that have tried to address parameter dependency by iteratively finding the best match for the parameters through multiple empirical runs leading to higher execution time [23], our approach is parameter-free.
4) We further provide a benchmark evaluation of the proposed approach on three publicly available datasets using four different state-of-the-art methods to demonstrate better performance. We supplement our qualitative and quantitative analysis with statistical analysis to establish the benefits of our proposed method.

## III. Preliminary Analysis of Highlight Pixels

Identifying and removing the highlight pixels requires adequate knowledge of the characteristics and distribution of highlight pixels. As inspired by [23], we evaluate the singular value distribution in III-A.

### A. Singular Value Distribution Of Highlight Images

**FIGURE 5.**
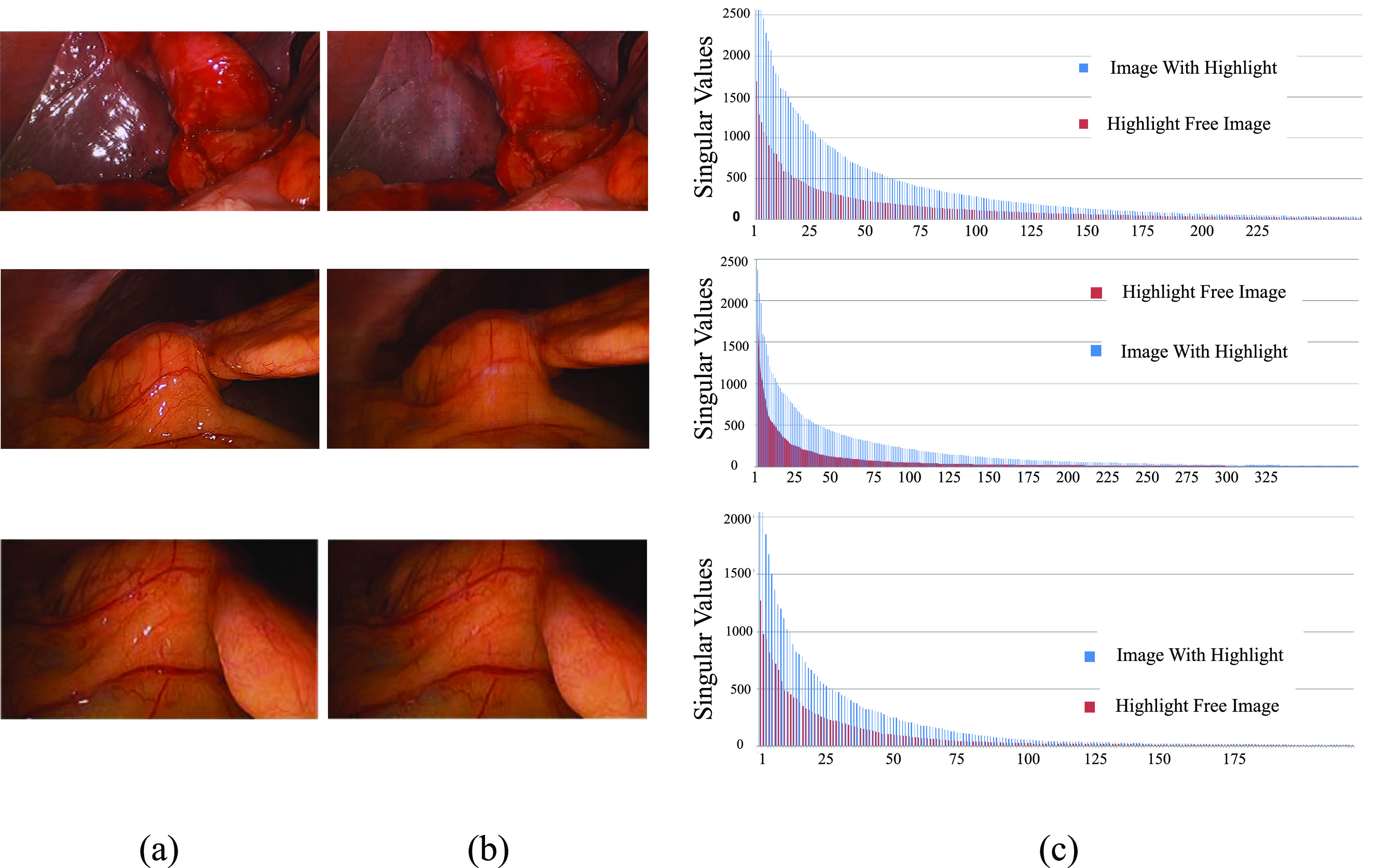
Singular value distribution for image with no highlight and highlight components (a) Images with highlight components (b) Highlight removed images and (c) Singular value distribution.

Fig. 5 depicts the study of the distribution of singular values in highlight-free images and highlight images [23]. It is observed in Fig. 5c, that the addition of highlight components into the highlight-free image, causes the distribution of singular values to change. New singular values are observed in the tail end of the distribution and magnitudes of upper singular values differ^1^^1^We refrain from presenting the singular values from the tail end for the sake of illustration.

In order to obtain a highlight-free image, the singular values may be modified by removing the lower singular values and reducing the magnitudes of the upper singular values. Since the distribution varies from image to image (Fig. 5(c)) and from one singular value to another, the amount of modification is difficult to predict. Further, the presence of noise factors in original image alters the distribution of singular values almost the same way as with the presence of specular reflection component. The chromatic characteristics detailed in Section III-B are used to determine the change in singular value distribution due to specular reflections. Applying chromatic characteristics specific to specular reflections can aid us in isolating the effect of specular reflections from the confounding effects of noise factors such as camera jitters and salt and pepper noise introducing uncertainty in estimating specular reflection robustly. A plausible solution is therefore to introduce an iterative method, which takes care of this uncertainty in the distribution by suitably applying the chromatic characteristics specific to sepcular reflections.

### B. Characteristics of Highlight Pixels

**FIGURE 6.**
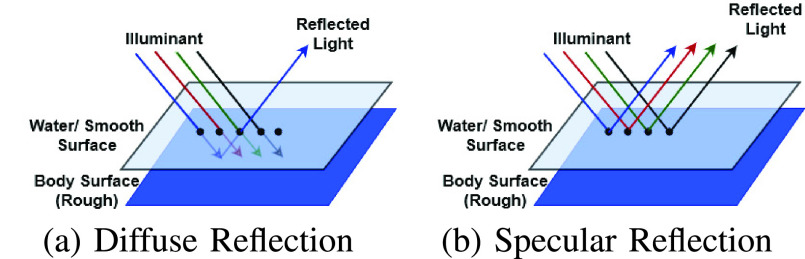
Reflection from the body surface and watery surface.

The gastrointestinal tract is usually covered by watery surfaces. When light is incident on a normal tissue surface, the reflected light contains the spectral component corresponding to the body surface. However, when the tissues are covered in a watery surface, almost all the spectral components are reflected back by this smooth Lambertian surface [30] as shown in Fig. 6. In the second case, the reflected light has the property of the illuminant used, which is generally having a large spectral width. As the spectral width increases, more whiteness is added to the image, which is similar to the dilution of the hue of the color. As the colors are diluted more, the saturation values become small.

A low absorption rate at the surface results in high-intensity values for specular reflections compared to the diffuse reflection rate. This reveals the next prominent characteristic of the specular reflection: high-intensity value. These two characteristics, low saturation, and high intensity, can be used to identify the highlight pixels supporting our hypothesis behind the proposed approach of using human vision-based processing of images.

## IV. Proposed Method

**FIGURE 7.**
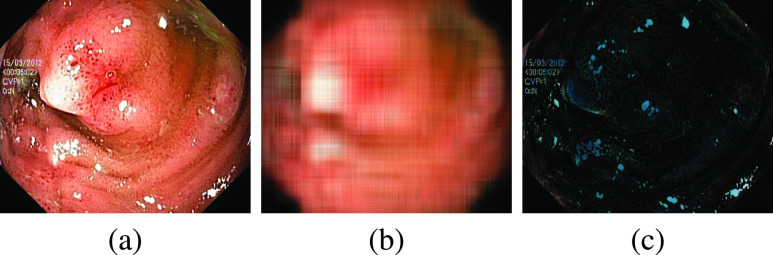
Conventional RPCA Decomposition (a) Original Image, (b) Low-Rank Component and (c) Sparse Component.

Our proposed approach is based on the idea of extracting the low-rank component embedded in the image, which is asserted to be highlight-free. We, therefore, intend to decompose the original image with highlight into a highlight-free pseudo-low-rank component and a sparse highlight component. However, the highlight component, which is generally sparse in nature may contain some useful information, some of which are vital for the identification of various pathologies. Conventional RPCA [28] approaches fail to work effectively with highlight removal as shown in Fig. 7, since the method tries to explore only the low-rank component hidden in the original image. This leads to the loss of vital information. An illustration of such a problem is provided in Fig. 7 where the resulting low-rank component is highly blurred losing key information but eliminating the highlight effectively. Care needs to be exercised to preserve key information by selectively processing the image not to affect the characteristics of the image around highlight pixels in the image. We, therefore, assert that a pseudo-low-rank component that contains no highlight, instead of generating a truly low-rank component, can eliminate the highlights without compromising the key information. We choose Singular Value Decomposition (SVD) [31] in our proposed approach for decomposing matrix as nearly low-rank and sparse components of the image as against traditional RPCA-based methods.

Our proposed method can be summarized in the following steps:

• Initially, a mask of highlight pixels is approximated by utilizing the properties of highlight pixels discussed in Section III-B.
• The low-rank component of the original image is then computed using the SVD approach. The remaining component is thus sparse, as the lower singular values correspond to them [28].
• At this point, the highlight component contains some required information and some highlight. The sparse component contains the distributed highlight and useful information in the form of sparse. We thus need to remove highlights from the low-rank component and add to the sparse component and useful information from the sparse component and re-insert them into the low-rank component, respectively.
• Hence, to distinguish the useful information and highlight in the sparse component, the characteristics of the highlight and the mask created are utilized. In order to remove the highlight further from the low-rank component, the extraction parameters are changed in the singular value decomposition.
• These two processes are repeated until no highlight is present in the low-rank component and no useful information is retained in the sparse component. The convergence of the algorithm is declared when the low-rank component is diffuse enough, at which point the sparse component provides the highlight component.

A detailed discussion of the various steps to obtain a Pseudo-Low-Rank component, which is the highlight-free image, is provided in the next section.

### A. Pseudo-Low-Rank and Highlight Decomposition

As argued earlier, the conventional RPCA framework fails to decompose the highlight image into highlight-free component and highlight component without losing the details. Hence this work proposes a modification in the decomposition process of the conventional RPCA framework. Let $\mathbf {X}$ denote the original image from which the highlight is to be removed. We intend to decompose $\mathbf {X}$ into a pseudo-low-rank component $\mathbf {L}$ and a highlight component $\mathbf {H}$, unlike conventional RPCA. Although the decomposition of the matrix $\mathbf {X}$ into a low-rank component and a sparse component is simple, the approximation of the sparse highlight component is not always true. The highlight components contribute significantly to the low-rank structure of the image itself. Again, some of the very essential features of the source image may get deleted from the low-rank image. So we introduce a new matrix decomposition method that utilizes the characteristics of the highlight pixels.

#### 1) Step 1: Mask Creation

A pixel is said to be a highlight pixel if its saturation value is less than a certain saturation threshold and its intensity value is greater than a certain threshold. Setting a hard threshold is challenging as the illumination differs for different images. To circumvent the problem, we adopt a soft threshold based on $\mathbf {S}$ and $\mathbf {V}$ channels in the $\mathbf {HSV}$ color space. Those pixels which have a saturation value lesser than the average value of the $\mathbf {S}$ channel and intensity value greater than the average value of $\mathbf {V}$ channel can be considered as highlight pixels. However, if an image does not contain any highlight pixels, the average values change accordingly and the thresholds will result in identifying incorrect pixels as highlight pixels leading to the failure of the method. We, therefore, propose an ensured minimum threshold value for the $\mathbf {S}$ channel as a hard threshold. The final soft threshold is computed as a minimum of the above two values. Similarly, a maximum value between the average intensity of the $\mathbf {V}$ channel and a hard threshold is chosen for computing the soft intensity threshold.

\begin{align*} \text {S}_{\tau} &= min\{\text {S}_{\text {mean}}, \:\text {S}_{\text {hard}} \} \\ \text {V}_{\tau} &= max\{\text {V}_{\text {mean}},\: \text {V}_{\text {hard}} \} \tag{1}\end{align*}

where, $\text {S}_{\text {mean}}$ and $\text {V}_{\text {mean}}$ are the mean value of $\mathbf {S}$ channel and $\mathbf {V}$ channel respectively. $\text {S}_{\text {hard}}$ and $\text {V}_{\text {hard}}$ are the user-defined fixed thresholds to control the illumination changes in the scene.

Now, a binary mask $\mathbf {M}$ is created with the same size as the input image $\mathbf {X}$. The mask has ones in the pixel positions corresponding to the pixels of the original image that satisfies the conditions for highlight discussed in Section III-B and is given in Eq. (2). For the $\textit {(i, j)}^{th}$ pixel,

\begin{equation*} \mathbf {M}(i, j) = 1,\quad if\;\; \mathbf {S}(i, j) < \text {S}_{\tau} \;\;and\;\; \mathbf {V}(i, j) > \text {V}_{\tau} \tag{2}\end{equation*}

**FIGURE 8.**
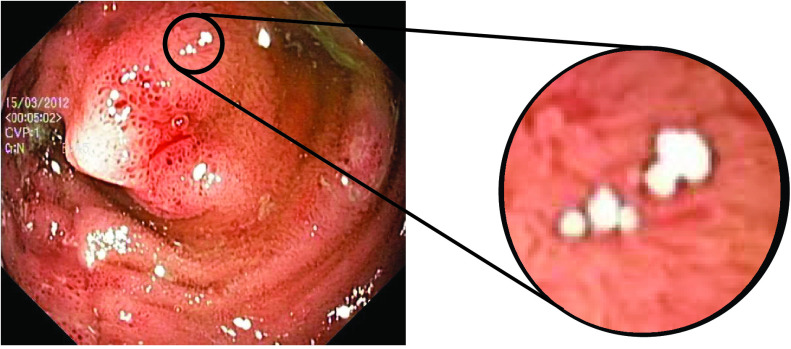
Boundary artifact around the highlight.

In addition to the highlight components, the presence of dark regions around the boundary of highlight pixels also need to be eliminated to avoid boundary inconsistencies. Close observation of the endoscopic images with highlight reveals that there is a small dark boundary associated with highlight regions. An illustration of this artifact is provided in Fig. 8 for an image from Kvasir Polyp dataset [14]. If such inconsistencies are not eliminated, the quality of the image after the highlight removal will be compromised.

**FIGURE 9.**
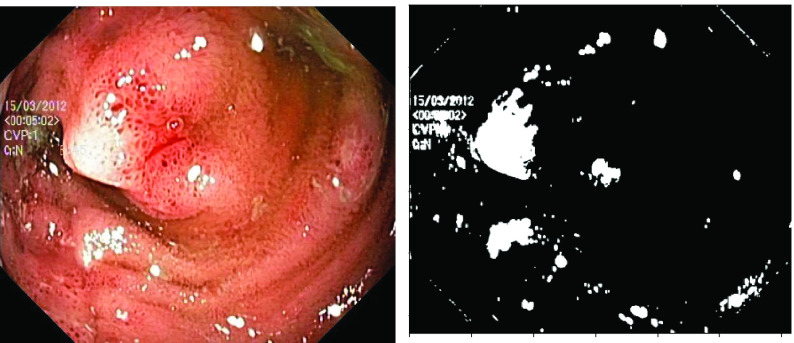
Highlight image and the corresponding binary Mask generated.

In order to remove these boundaries which have different characteristics than that of highlights, the mask is to be recalculated. A morphological dilation operation is performed on the available mask to obtain a new mask with a suitable structuring element^2^. A typical mask corresponds to an endoscopic image from the Kvasir Polyp dataset [14] is given in Fig. 9.^2^We have used a structuring element of $7\times 7$ pixels.

#### 2) Step 2: Extracting the Low-Rank Component

**FIGURE 10.**
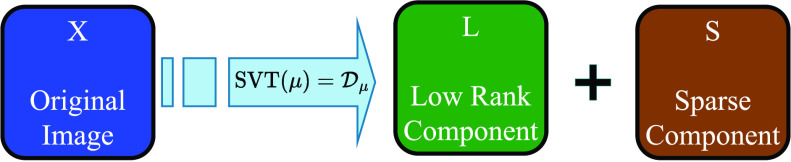
Low rank + Sparse Decomposition.


Once the mask is created, iterative decomposition procedure can be initiated. Initially the Singular Value Thresholding (SVT) operator [32] is applied on the original image to obtain a low-rank component $\mathbf {L}$ and a sparse component $\mathbf {H}$ using Eq. (3) as illustrated in Fig. 10.

\begin{align*} \mathbf {L} &= \mathcal {D}_{\mu }(\mathbf {X}) \\ \mathbf {S} &= \mathbf {X} - \mathbf {L} \tag{3}\end{align*}

where, $\mathcal {D}_{\mu }$ is the SVT operator with parameter $\mu $. The SVT operator is defined as,

\begin{equation*} \mathcal {D}_{\mu} (\mathbf {X}) = \mathbf {U}\mathcal {S}_{\frac {1}{\mu }}(\Sigma)\mathbf {V}^{T} \tag{4}\end{equation*}

where, $\mathbf {X}$ = $\mathbf {U}\Sigma \mathbf {V}^{T}$ is the SVD of the matrix $\mathbf {X}$, with $\Sigma $ being the singular value matrix. $\mathcal {S}_{\tau} (\mathbf {.})$ is the soft thresholding operator defined as,

\begin{equation*} \mathcal {S}_{\tau} (\mathbf {X}) = [\mathcal {S}_{\tau} (x), \forall {x}\in \mathbf {X}] \tag{5}\end{equation*}

\begin{equation*}\mathcal {S}_{\tau} (x) = sgn(x) \times \text {max}\{ x\;-\;\tau \;,\;0\}\end{equation*}

The value of $\mu $ is selected such that a large number of singular values towards the tail end of the distribution is made to zero in the SVT operation. This helps to extract maximum highlight components from the original image $\mathbf {X}$. The resulting low-rank component still contains some highlight regions and the whole image will be blurred out. The residue matrix $\mathbf {X} - \mathbf {L}$ is the sparse component and contains both highlight and useful information which are sparse in nature.

#### 3) Step 3: Extracting the Highlight Components

The sparse matrix $\mathbf {S}$ is composed of highlight component and useful information which are sparse in nature. In order to retrieve the useful information from the sparse matrix, the mask prepared in *Step 1* can be used. Since the positions of the useful information are mutually exclusive with that of the highlight pixels, multiplying the sparse matrix with the mask will provide highlight component only.

\begin{equation*} \mathbf {H}\;=\;\mathbf {S} \otimes \mathbf {M} \tag{6}\end{equation*}

where, $\otimes $ represents the pixel-wise multiplication and $\mathbf {M}$ is the mask created. The operation is illustrated in Fig. 12.

**FIGURE 11.**
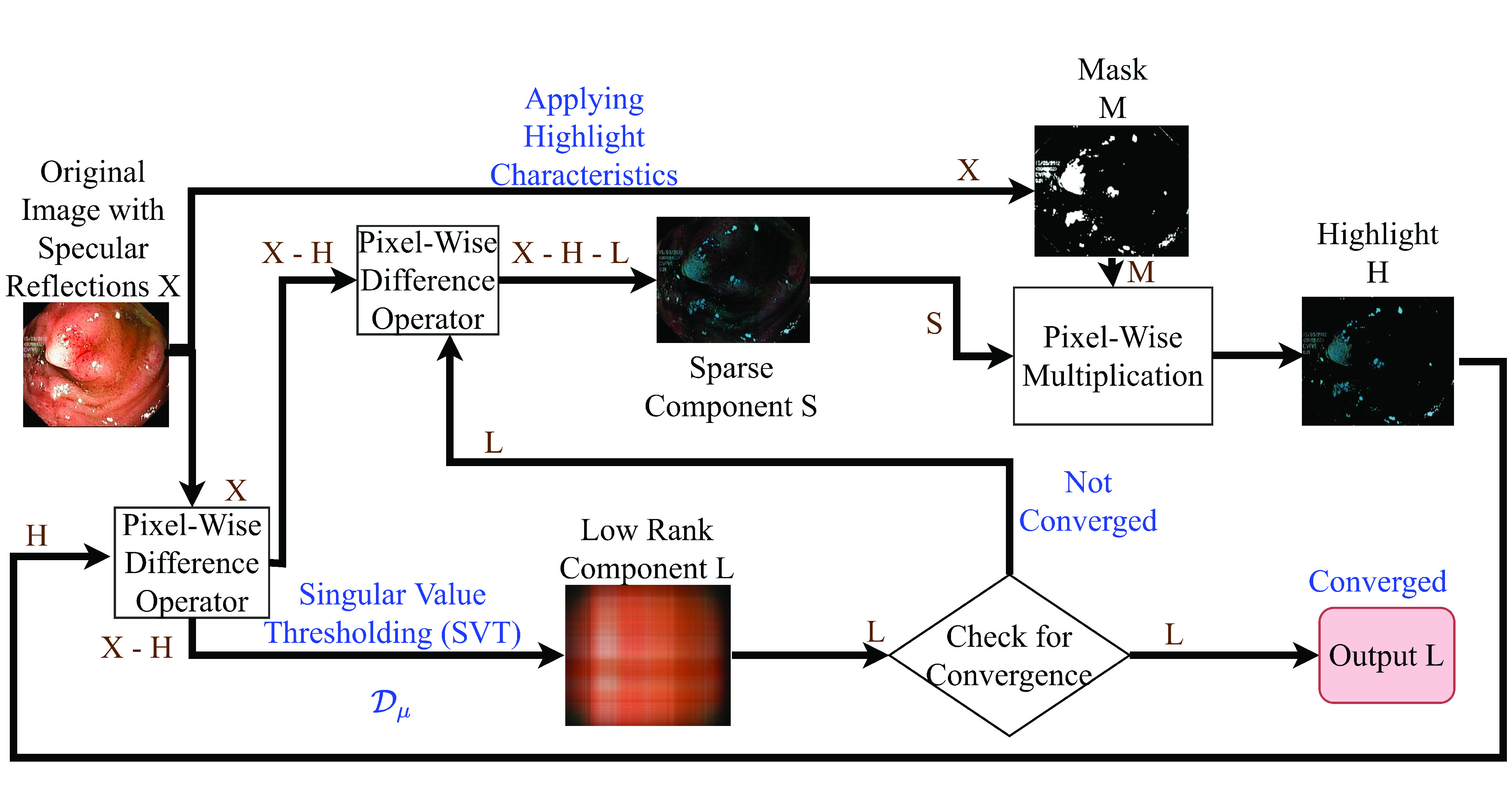
Various steps in the proposed method.

**FIGURE 12.**
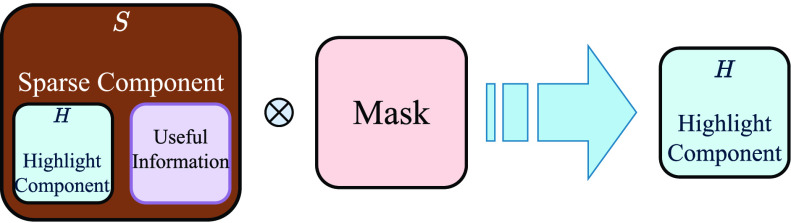
Extraction of highlight component from the Sparse component.

The remaining part of the sparse matrix, which is the residue matrix, represents the useful information.

#### 4) Step 4: Iteration

If the residue matrix $\mathbf {S} - \mathbf {H}$ after the multiplication process is significantly high, *step 2* and *step 3* are repeated until this quantity becomes less than a threshold as per the convergence criteria. Each iteration begins with an augmented image $\mathbf {X} - \mathbf {H}$, in which already extracted highlight components are removed. While the highlight component which is mostly sparse is removed, there is no guarantee of eliminating all highlight regions. The remaining $\mathbf {X} - \mathbf {H}$ can therefore be called the pseudo-low-rank component as opposed to the true low-rank component. Further, the SVT variable $\mu $ is updated in each iteration by multiplying with a fixed updating parameter $\lambda $. The complete iteration procedure is as follows.

\begin{align*} \mathbf {L}&= \mathcal {D}_{\mu }(\mathbf {X} - \mathbf {H}) \\ \mathbf {H} &= (\mathbf {X} - \mathbf {L}) \otimes \mathbf {M} \\ \mu &= \lambda \mu \tag{7}\end{align*}

The iteration continues until the residue matrix $\mathbf {S}$ - $\mathbf {H}$ = $\mathbf {X}$ - $\mathbf {L}$ - $\mathbf {H}$ is significantly small. The convergence condition is formulated as,

\begin{equation*} \|\mathbf {X} - \mathbf {L} -\mathbf {H}\|^{2}_{F} \leq \zeta \tag{8}\end{equation*}

where $\|{.}\|^{2}_{F}$ is the Frobenius norm and $\zeta $ is the threshold for convergence.

The overall process used to obtain the highlight-free image from the original endoscopic image is described in Fig. 11. The figure depicts the results obtained at various levels of the algorithm with respect to one image from the Kvasir Polyp dataset [14]. Algorithm 1 depicts the complete steps involved in the proposed method.

Algorithm 1 **Removing Highlight components from WCE images**
1 **Input:** X $\in \,\,\mathcal {R}^{w\times h\times 3}$
2 **Output:** L $\in \,\,\mathcal {R}^{w \times h\times 3}$
1 **Initialize:** L, H = 0, $\mu $ = 0.0006, $\lambda $ = 1.2
4 Read Image to **X**.
5 Calculate $\text {S}_{\tau }$ and $\text {V}_{\tau} $ using Eq. (1).
6 Generate the binary Mask **M** using Eq. (2).
7 Update **L** & **H** according to Eq. (7).
8 Check for convergence. The condition is $\|\textbf {X}-\textbf {L}-\textbf {H}\|_{F}^{2} < = \zeta $. If the condition is satisfied, the Algorithm stops and **L** gives the highlight-free image. Else repeat from Step 7.

## V. Experimental Results and Discussions

The proposed algorithm is tested on three different datasets to check for the generalizability of the algorithm. Our aim is to assess the proposed algorithm’s generalizability by applying it to diverse datasets containing different types of modalities and including both normal and pathology sets. To achieve this, the Kvasir dataset from Simula Research Laboratory [14] and the Kvasir Capsule dataset [15] are utilized. We have selected the datasets to ensure the images of different resolution and quality works equally under the proposed algorithm.

The Kvasir dataset is an annotated collection of images used for identifying pathology, comprising eight different image classes. For evaluation of the proposed work, two image classes from the Kvasir dataset are selected. The algorithm is tested on the ‘Polyp’ class, as polyp detection and segmentation are currently prominent areas of research in endoscopy. To verify its effectiveness on normal and pathology images alike, the ‘Normal-Pylorus’ class is randomly selected from the three available normal classes. These classes will be referred to as the Kvasir Polyp dataset and Kvasir Normal-Pylorus dataset from now onwards. 500 images with specular reflections from both classes are selected randomly and are used in the evaluation process. We also use the Kvasir Capsule dataset [15], created using PillCam Capsule Endoscopy System which contains 47,238 labeled images and 117 videos. We have identified 330 images with significant specular reflections and are selected to evaluate the proposed method^3^. The images are selected to ensure that they contain specular reflections in various amounts for each dataset. Since this method is developed to remove specular reflections from endoscopic images for which the ground truth is usually unavailable, the method is compared with the state-of-the-art highlight removal algorithms based on classical computer vision methods rather than the deep learning methods.^3^All the algorithms were run on Python 3.9 on Windows 11 platform with intel(R) Core(TM) i7-10700K CPU @3.79GHz and 32GB RAM.

To holistically evaluate the proposed approach, we compute Structural Similarity Index Measure (SSIM) [33], the percentage of highlights removed Eq. (9), and Coefficient of Variation (CoV) [34] as explained in the subsequent sections. We compare our proposed algorithm with Adaptive RPCA proposed by Li et al. [23], inpainting technique by Arnold et al. [16], NONRPCA method [35] and RPCA method [28] to establish the benefits of our proposed approach.

**FIGURE 13.**
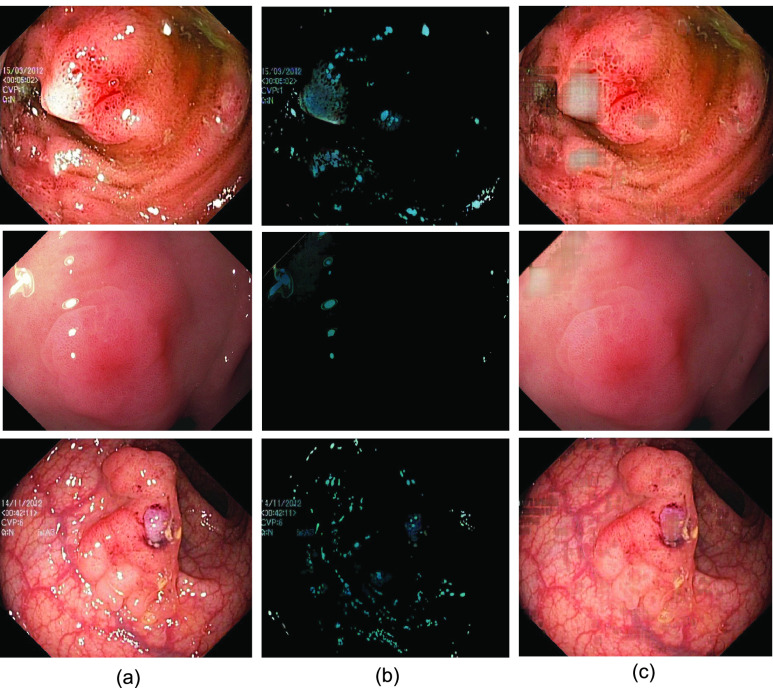
Result of Applying proposed method to Kvasir Polyp dataset. (a) Original Image, (b) Highlight Component (c) pseudo-Low-Rank Component.

**FIGURE 14.**
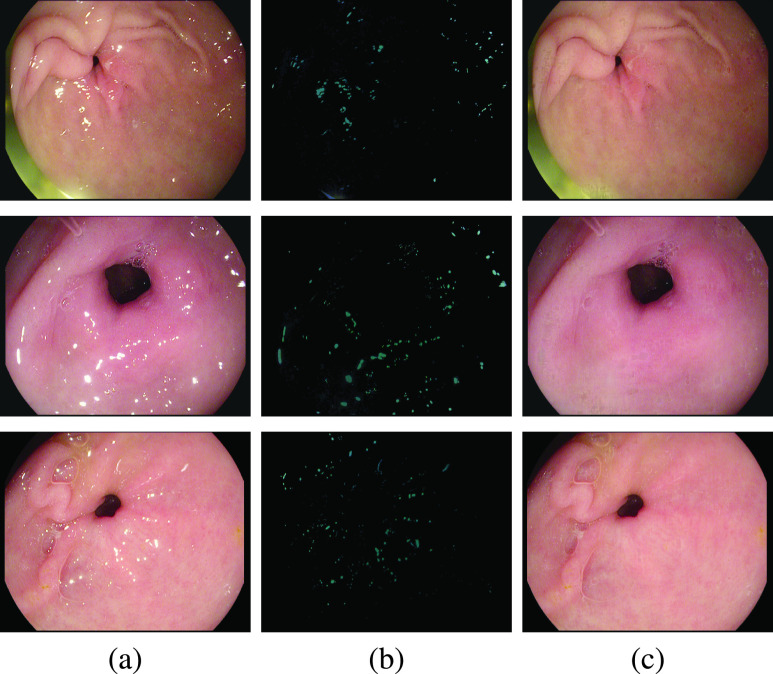
Result of Applying proposed method to Kvasir Normal-Pylorus dataset. (a) Original Image, (b) Highlight Component (c) pseudo-Low-Rank Component.

**FIGURE 15.**
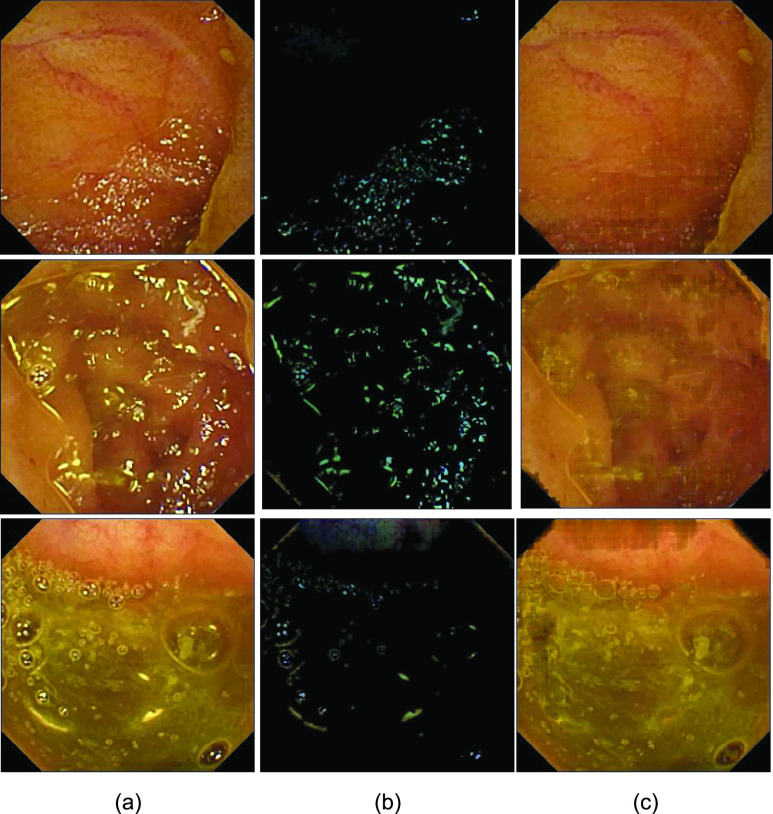
Result of Applying proposed method to Kvasir Capsule dataset. (a) Original Image, (b) Highlight Component (c) pseudo-Low-Rank Component.

Before delving into quantitative analysis, we present a qualitative illustration of the proposed approach as shown in Fig. 13, Fig. 14 and Fig. 15 corresponding to Kvasir Polyp [14], Kvasir Normal-Pylorus [14] and Kvasir Capsule [15] datasets, respectively. In each of the figures, (a) represents the original image with highlight pixels, (b) represents the highlight component, and (c) represents the highlight-free pseudo-low-rank component.

### A. Visual Comparison with Other Highlight Removal Algorithms

**FIGURE 16.**
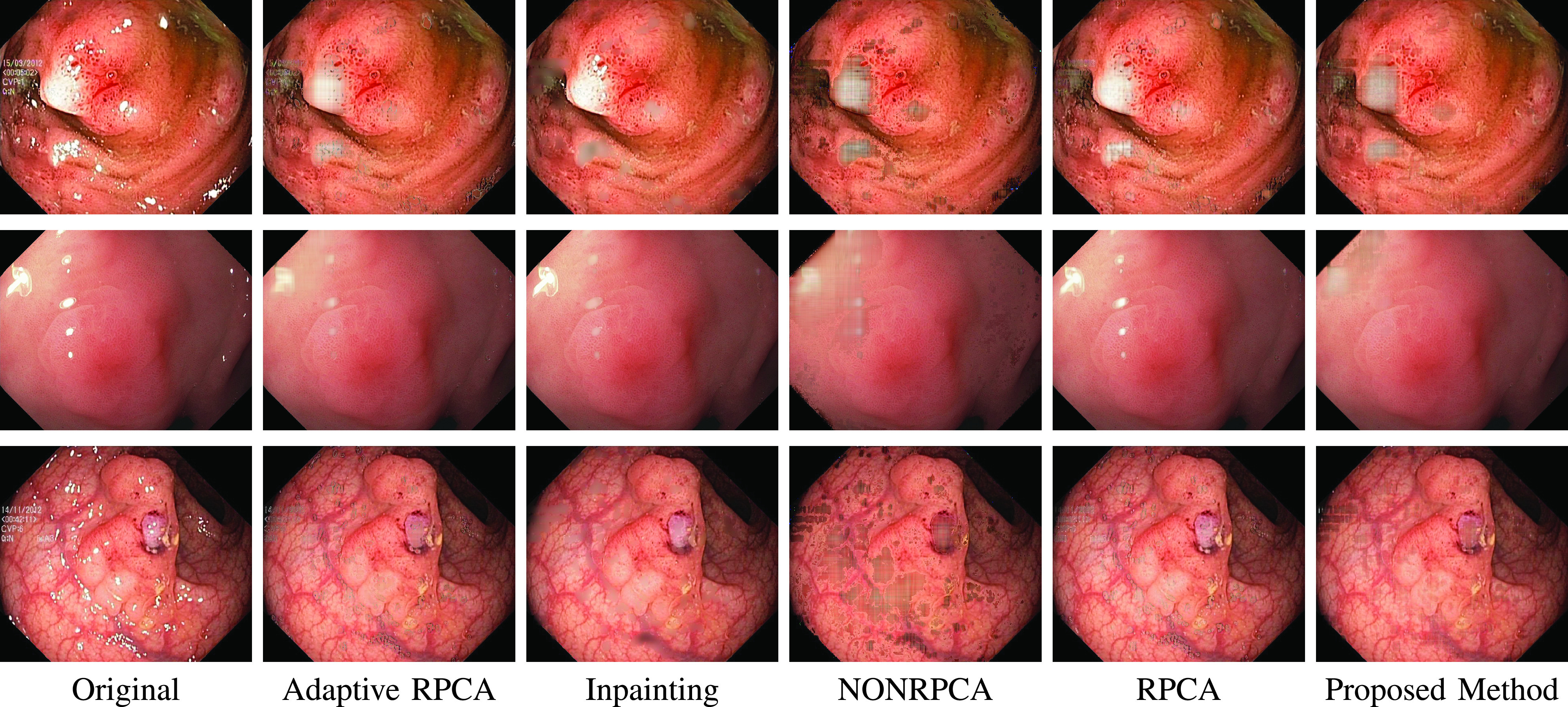
Highlight Removed images from Kvasir Polyp dataset using various algorithms. From left to right: Original image, Adaptive RPCA [23], Inpainting [16], NONRPCA [35], RPCA [28] and proposed method.

**FIGURE 17.**
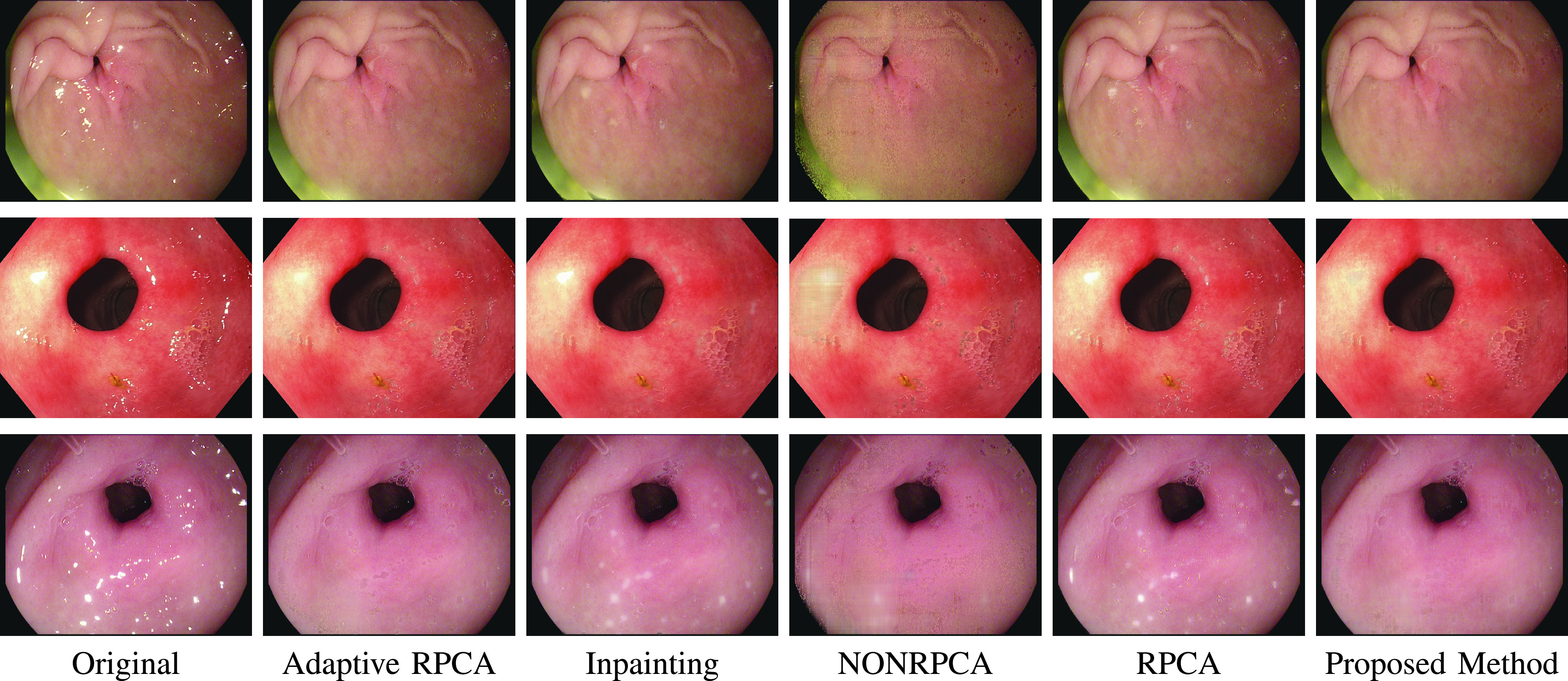
Highlight Removed images from Kvasir Normal-Pylorus dataset using various algorithms. From left to right: Original image, Adaptive RPCA [23], Inpainting [16], NONRPCA [35], RPCA [28] and proposed method.

**FIGURE 18.**
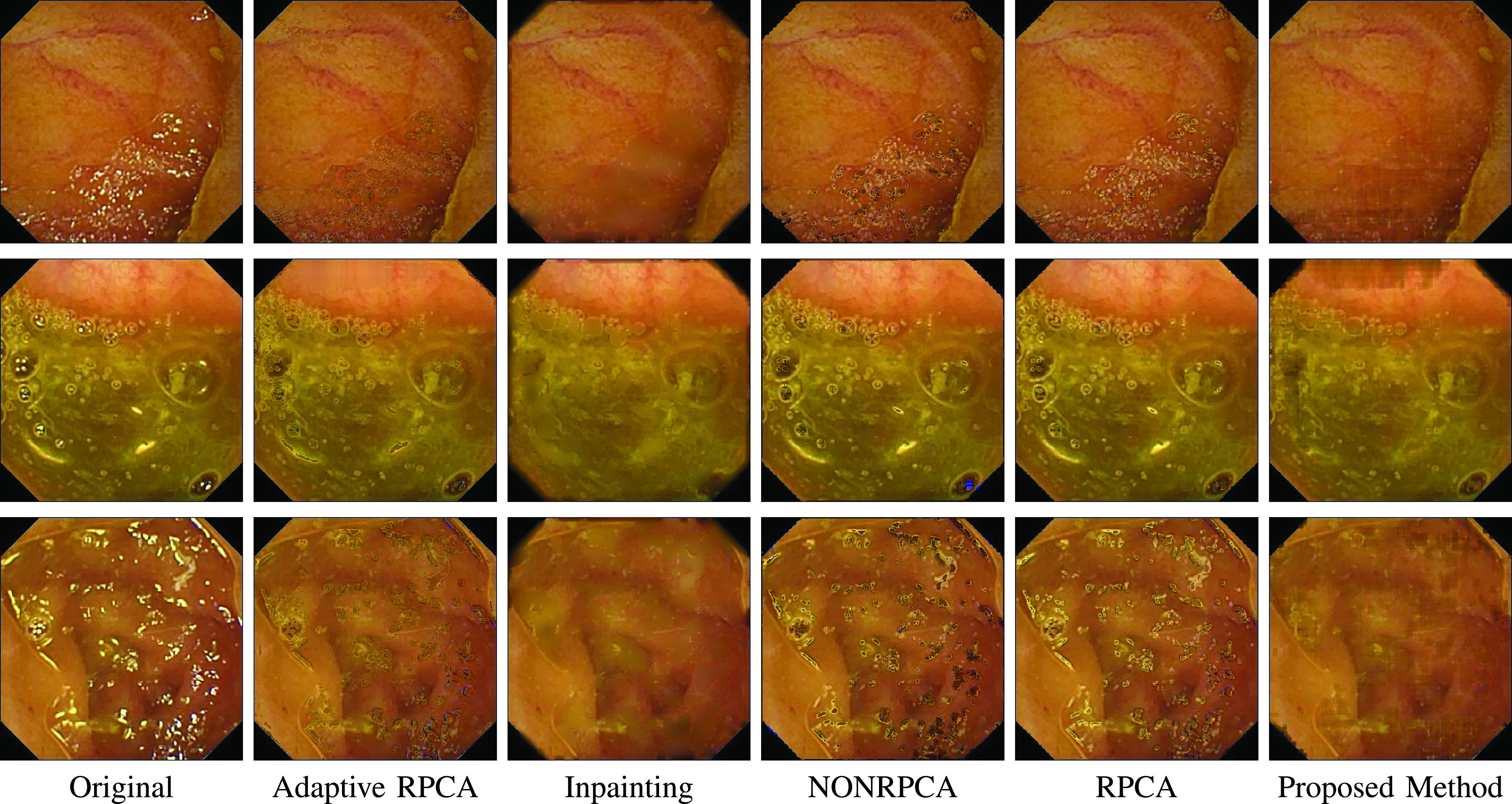
Highlight Removed images from Kvasir Polyp dataset using various algorithms. From left to right: Original image, Adaptive RPCA [23], Inpainting [16], NONRPCA [35], RPCA [28] and proposed method.

A visual comparison of the results is provided in Fig. 16, Fig. 17 and Fig. 18 for Kvasir Polyp, Kvasir Normal-Pylorus and Kvasir Capsule datasets respectively. Adaptive RPCA [23] iteratively finds the decomposition parameter $\lambda $ that best suits to remove all the highlight pixels from the original image. The algorithm works well unless the highlight component contributes significantly to the low-rank component of the image. A significant deficiency with Adaptive RPCA [23] is the impact of parameter $\lambda $, resulting in very dark colors at the positions of highlight regions when it tries to remove the highlight component largely rooted in the original image. The first and second rows in Fig. 16 evidently illustrated the inability of the algorithm to remove the highlight component. Close observation further reveals that the boundary artifacts present around the highlight regions are also intact in all the highlight-removed images.

The results of the inpainting method [16], specifically for the Kvasir Normal-Pylorus dataset, reveals that the inpainted regions do not blend with its neighborhood region although the highlight pixels are removed. NONRPCA [35] method also suffers from these drawbacks. When coming to RPCA [28], keeping the parameter $\lambda $ constant is not going to give good results as the depth of sparseness is different for different images. In contrast, the proposed method takes care of these drawbacks and the results obtained are promising. With a single parameter setting, the algorithm works well for all the three datasets considered as indicated in Fig. 16, Fig. 17 and Fig. 18.

### B. Quantitative Comparison of the Results

To further quantify the comparison results, three metrics such as the Structural Similarity Index Measure (SSIM), the percentage of highlights remaining and the Coefficient of Variation (CoV) are computed whose analysis is presented further.

#### 1) Comparison of SSIM

To evaluate the efficiency of the proposed method, SSIM is calculated between the initial mask generated for the proposed method and the highlight component obtained after the decomposition for various methods taken for comparison. The initial mask is considered the required highlight component. Table 1 gives the statistical distribution of the SSIM scores computed for the Kvasir Polyp [14], Kvasir Normal-Pylorus [14] and Kvasir Capsule [15] datasets. As there is no highlight component available from the inpainting method, the SSIM scores are not calculated for inpainting technique by Arnold et al. [16].

**TABLE 1:** Statistical distribution of SSIM scores. The scores for Inpainting are not calculated due to the unavailability of actual ground truth. *Note - The higher the scores, the better the method

Dataset	Adaptive RPCA $(\downarrow)$	NONRPCA $(\downarrow)$	RPCA $(\downarrow)$	Proposed $(\uparrow)$
Kvasir Polyp	0.864 ± 0.05	0.1164 ± 0.0353	0.3368 ± 0.0562	0.8745 ± 0.0481
Kvasir Normal-Pylorus	0.8582 ± 0.0802	0.1488 ± 0.0764	0.4113 ± 0.0949	0.8724 ± 0.0813
Kvasir Capsule	0.7842 ± 0.0934	0.0481 ± 0.0106	0.226 ± 0.0315	0.8146 ± 0.0963

The higher the SSIM, the better the quality of the highlight removed. As evident from the results in Table 1, the proposed approach results in a high SSIM indicating the performance of the proposed approach in removing the highlight pixels irrespective of the sparse nature of the highlight component. SSIM scores for the proposed method and for the Adaptive RPCA method are comparable. But the scores for NONRPCA and RPCA methods are very poor as indicated.

#### 2) Percentage of Highlights Remaining

The percentage of highlights remaining is calculated as,

\begin{equation*} H_{r} = \frac {N_{h}}{N_{t}}\times 100 \tag{9}\end{equation*}

where $H_{r}$ is the percentage of highlights remaining, $N_{h}$ is the number of highlight pixels in the image after highlight removal and $N_{t}$ is the total number of pixels in the original image.

**FIGURE 19.**
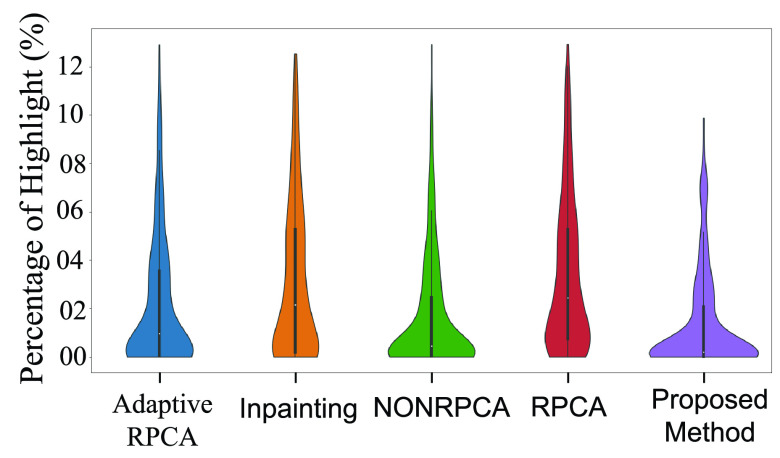
Statistical distribution of Percentage of highlight remaining in Kvasir Polyp dataset.

**FIGURE 20.**
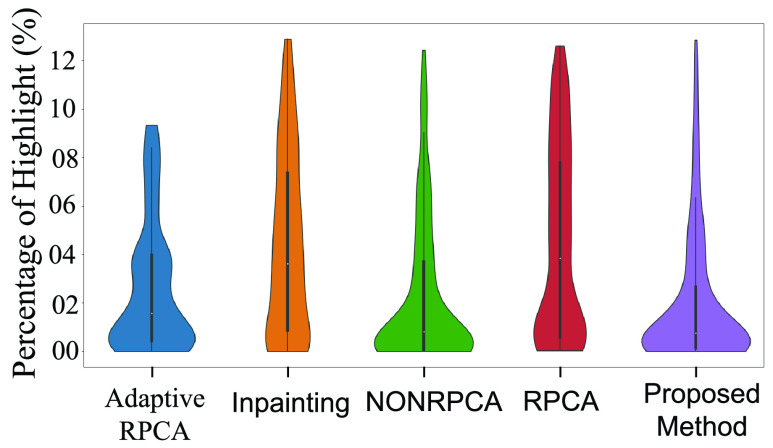
Statistical distribution of Percentage of highlight remaining in Kvasir Normal-Pylorus Dataset.

**FIGURE 21.**
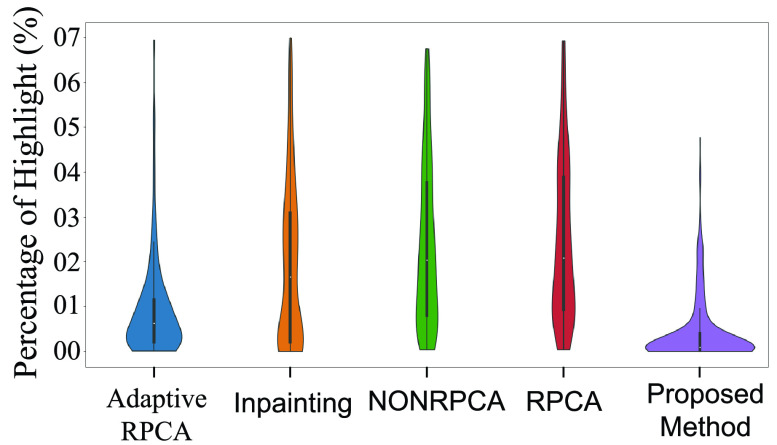
Statistical distribution of Percentage of highlight remaining in Kvasir Capsule Dataset.

Table 2 shows the distribution of the percentage of highlights for various algorithms discussed in the previous section. Clearly, the proposed method outperforms all the compared techniques on all three datasets. In addition to Table 2, we also present Violin-plots to demonstrate the statistical significance analysis as shown in Fig. 19, Fig. 20 and Fig. 21 for the three datasets. Fig. 19 details the distribution of the percentage of highlights remaining in the Kvasir Polyp dataset after the application of various algorithms. The position of the median for the proposed method lies below all the other medians. Further, the point of the maximum width of the violin plot for the proposed method is lower than other methods. From Fig. 19, it is obvious that the standard deviation for the proposed method is minimum when compared to other methods. This shows the stability of the proposed method. Similar trends can be observed for both the Kvasir Normal-Pylorus dataset and Kvasir Capsule dataset as indicated in Fig. 20 and Fig. 21, respectively.

**TABLE 2:** Statistical distribution of Percentage of Highlight remaining ( $H_{r}$)(%) computed using Eq. (9). (± represents deviations computed at 95% confidence interval (CI)). *Note - The lower the scores, the better the method

Dataset	Adaptive RPCA $(\uparrow)$	Inpainting $(\uparrow)$	NONRPCA $(\uparrow)$	RPCA $(\uparrow)$	Proposed $(\downarrow)$
Kvasir Polyp	2.2866 ± 0.2608	3.3744 ± 0.3142	1.7881 ± 0.2313	3.6325 ± 0.3081	1.3939 ± 0.1890
Kvasir Normal-Pylorus	3.9889 ± 0.5032	5.6336 ± 0.4778	2.9827 ± 0.4366	5.5561 ± 0.447	2.5333 ± 0.3639
Kvasir Capsule	1.3589 ± 0.2336	2.8722 ± 0.3718	2.9878 ± 0.2851	3.3308 ± 0.3427	0.5359 ± 0.0998

The statistical significance of the percentage of highlight removed is deduced by performing a one-way ANOVA test [36] with a significance level set at 0.05 and is presented in Table 3. The table clearly demonstrates the significance as the $P$-Values are very small. The only case where the two distributions are almost the same is between the proposed method and the NONRPCA for the dataset Kvasir Normal-Pylorus. This is consistent with the observations made in Fig. 20.

**TABLE 3:** Statistical significance analysis using one-way ANOVA test for the percentage of highlight removed metric and CoV metric. The significance level was established at 0.05

Dataset	Approach	$P$-Value (Percentage of Highlight)	$P$-Value (CoV)
Kvasir Polyp	Adaptive RPCA	$3.97\times 10^{-08}$	$3.52\times 10^{-02}$
	Inpainting	$1.26\times 10^{-24}$	$2.85\times 10^{-10}$
	NONRPCA	0.015	0.697
	RPCA	$6.42\times 10^{-32}$	$3.54\times 10^{-03}$
Kvasir Normal-Pylorus	Adaptive RPCA	$2.787\times 10^{-04}$	$4.08\times 10^{-13}$
	Inpainting	$1.72\times 10^{-28}$	$4.57\times 10^{-07}$
	NONRPCA	0.2140	$4.72\times 10^{-03}$
	RPCA	$3.256\times 10^{-28}$	$6.89\times 10^{-02}$
Kvasir Capsule	Adaptive RPCA	$8.78\times 10^{-11}$	$7.92\times 10^{-07}$
	Inpainting	$8.02\times 10^{-36}$	$2.34\times 10^{-06}$
	NONRPCA	$1.18\times 10^{-55}$	$1.95\times 10^{-21}$
	RPCA	$1.56\times 10^{-61}$	$6.16\times 10^{-06}$

#### 3) Coefficient of Variation (CoV)

Although the highlights are removed completely, the reconstructed regions should blend with their surroundings to not compromise the quality of the image. We, therefore, employ the Coefficient of Variation (CoV) to measure the image quality in the vicinity of the highlight regions. It is the ratio between the standard deviation $\sigma $ and mean of the region $\mu $, i.e. [34],

\begin{equation*} CoV = \dfrac {\sigma }{\mu } \tag{10}\end{equation*}

**FIGURE 22.**
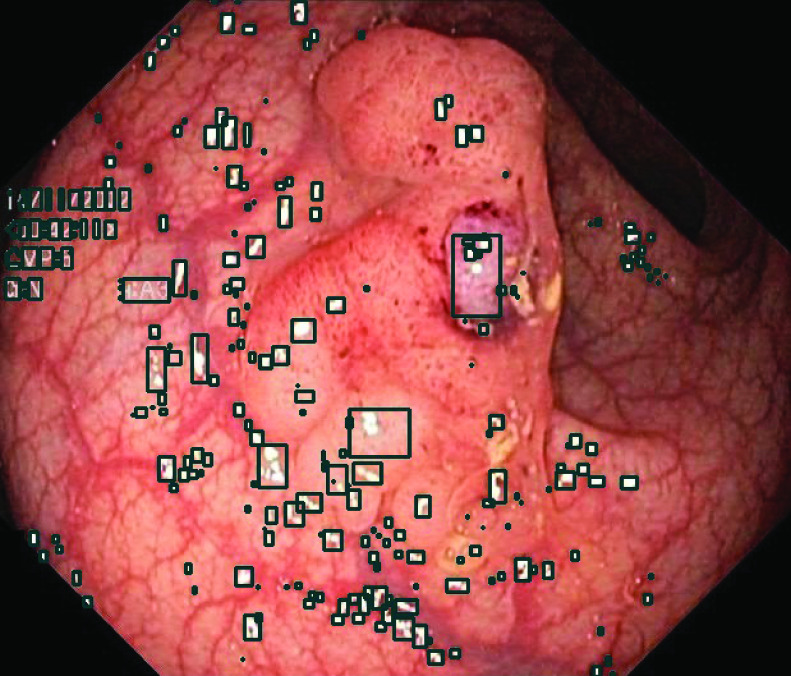
Bounding boxes showing the neighborhood of highlight regions for calculation of CoV.

CoV is calculated for the neighborhood of all the highlight regions as shown in Fig. 22 and the CoV score for the whole image is obtained as the mean value for all the regions. If the reconstructed regions blend with their surroundings, the normalized standard deviation is expected to be small. On the other hand, if the reconstructed regions fall far away from the neighborhood values, the normalized standard deviation and the CoV score will be high.

The CoV values are calculated on all three datasets, Kvasir Polyp, Kvasir Capsule and Kvasir Normal-Pylorus, and similar values are computed for state-of-the-art methods as presented in Table 4. The proposed method shows good blending of reconstructed regions with their surroundings as compared to other state-of-the-art methods used for comparison. This is in accordance with the visual comparison results from Fig. 16, Fig. 17 and Fig. 18. The quantitative significant analyses are presented in Table 3 with the help of one way ANOVA test at significant level (p<0.05). The $P$-Values are much smaller than the significance level set. The scores for the original image is the average CoV for the highlight image. As the highlight regions’ chromatic distribution is completely different from the chromatic distribution of the surroundings, the CoV score will be high for images with highlight as indicated in Table 4.

**TABLE 4:** Statistical distribution of Coefficient of Variation (CoV) *Note - The lower the scores, the better the method

Dataset	Original	Adaptive RPCA	Inpainting	NONRPCA	RPCA	Proposed
Kvasir Polyp	0.257 ± 0.045	0.206 ± 0.05	0.244 ± 0.088	0.215 ± 0.089	0.223 ± 0.051	0.212 ± 0.062
Kvasir Normal-Pylorus	0.185 ± 0.072	0.158 ± 0.043	0.154 ± 0.049	0.147 ± 0.049	0.139 ± 0.055	0.139 ± 0.039
Kvasir Capsule	0.357 ± 0.036	0.343 ± 0.061	0.338 ± 0.044	0.352 ± 0.034	0.338 ± 0.038	0.325 ± 0.035

### C. Limitations of the Proposed Method

**FIGURE 23.**
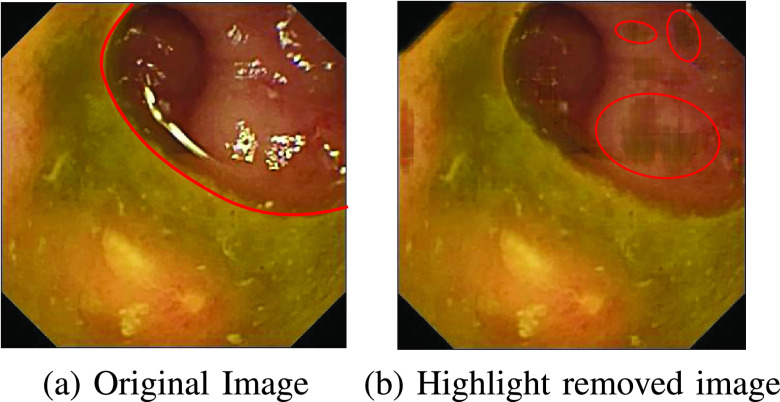
Illustration of low blending with the near neighborhood of the reconstructed highlight regions. The regions inside the red lines show similarity to other regions in the whole image in the right-hand image.

Although the method works efficiently on various datasets, it suffers from some demerits. Because of the global nature of the singular value thresholding operation and low rank-based extraction, sometimes it is impossible to blend perfectly with the near neighborhood of the reconstructed region as shown in Fig. 23. If the original image can be seen as two independent areas separated by the illustrative red line, we can see the major portion of the area in the image is covered by the region below the red line. Our method estimates the low rank based on the whole image and the lower portion of the image (below the red line) contributes significantly to estimating the low-rank component of the image. As the major contributor to the image is the lower region, the reconstructed sections have chromatic similarity to the lower portion than the upper portion. This leads to the degradation in the blending of the reconstructed regions and hence the value of the Coefficient Of Variation.

**FIGURE 24.**
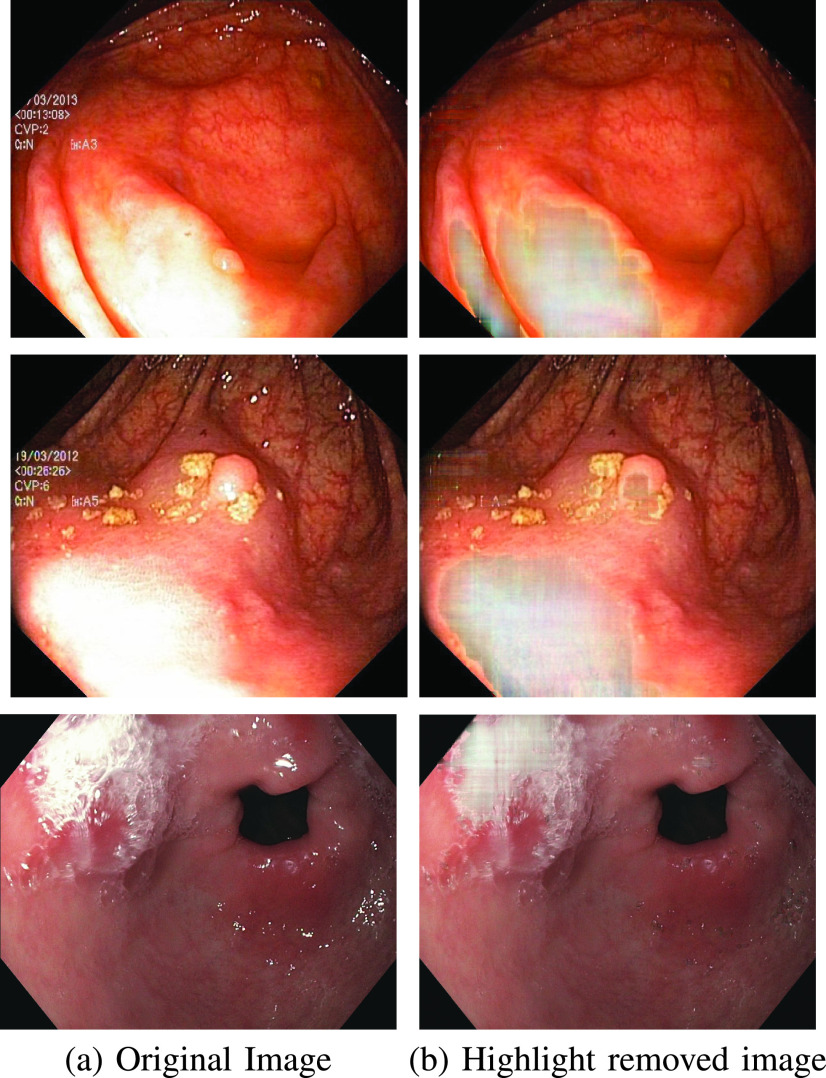
Illustration of failed cases of overexposed images when applying the proposed method.

Further, if the overexposed regions cover a large portion of the image, as shown in column one in Fig. 24, our approach becomes restricted. The contribution of high-intensity regions to the low-rank component is very high and as our algorithm depends on the contribution of each feature to the low-rank component of the image, the overexposed regions can contribute significantly to the low-rank component. Column two in Fig. 24 shows the result of the application of our method to some overexposed images resulting in poor highlight-free images.

### D. Potential Future Works

Conditional GANs can be used to remove highlights from endoscopic images by conditioning the generator network on the input image with highlights as well as an additional condition that describes the highlight regions in the image. To train the conditional GAN for highlight removal, a dataset of endoscopic images with corresponding highlight region conditions should be used. The network is trained to minimize the difference between the generated output image and the ground truth image without highlights, while also ensuring that the discriminator can distinguish between real and fake images. Once the network is trained, it can remove highlights from new endoscopic images by conditioning on the highlight region condition. However, the availability of large-scale data for training a cGAN still remains an open problem.

## VI. Conclusion

The proposed algorithm decomposes the original highlight image into a pseudo-low-rank component and a highlight component. The highlight-free image, which is the desired output, will then be the pseudo-low-rank component. The paper exploits the non-sparse nature of the highlight component to propose a generalizable method. The method does not require any parameter setting, and thereby no fine-tuning is involved making it a parameter-free approach. Our results on three different public datasets suggest the promising nature of the proposed algorithm. Even though the proposed algorithm works well for various datasets, the reconstructed regions do not completely blend with their surroundings. We hypothesize this to be a result of the global nature of the low-rank decomposition. Further, this method does not recover the actual information behind the highlight region. In future works, the idea proposed in the paper can be extended to include the information from temporal frames to recover the actual information behind the highlight regions.
